# A Randomised Controlled Trial to Assess the Efficacy of Dihydroartemisinin-Piperaquine for the Treatment of Uncomplicated Falciparum Malaria in Peru

**DOI:** 10.1371/journal.pone.0001101

**Published:** 2007-10-31

**Authors:** Tanilu Grande, Andrea Bernasconi, Annette Erhart, Dioni Gamboa, Martin Casapia, Christopher Delgado, Kathy Torres, Caterina Fanello, Alejandro Llanos-Cuentas, Umberto D'Alessandro

**Affiliations:** 1 Institute of Ttropical Medicine - Alexander von Humboldt (ITM-AvH), Universidad Peruana Cayetano Heredia, Lima, Peru; 2 Prince Leopold Institute of Tropical Medicine, Antwerp, Belgium; 3 Dirección Regional de Salud Loreto, Ministerio de Salud del Peru, Lima, Peru; 4 London School of Tropical Medicine and Hygiene, London, United Kingdom; Royal Melbourne Hospital, Australia

## Abstract

**Background:**

Multi-drug resistant falciparum malaria is an important health problem in the Peruvian Amazon region. We carried out a randomised open label clinical trial comparing mefloquine-artesunate, the current first line treatment in this region, with dihydroartemisinin-piperaquine.

**Methods and Findings:**

Between July 2003 and July 2005, 522 patients with *P. falciparum* uncomplicated malaria were recruited, randomized (260 with mefloquine-artesunate and 262 with dihydroartemisinin-piperaquine), treated and followed up for 63 days. PCR-adjusted adequate clinical and parasitological response, estimated by Kaplan Meier survival and *Per Protocol* analysis, was extremely high for both drugs (99.6% for mefloquine-artesunate and 98.4% and for dihydroartemisinin-piperaquine) (RR: 0.99, 95%CI [0.97−1.01], Fisher Exact p = 0.21). All recrudescences were late parasitological failures. Overall, gametocytes were cleared faster in the mefloquine-artesunate group (28 *vs* 35 days) and new gametocytes tended to appear more frequently in patients treated with dihydroartemisinin-piperaquine (day 7: 8 (3.6%) *vs* 2 (0.9%), RR: 3.84, 95%CI [0.82–17.87]). Adverse events such as anxiety and insomnia were significantly more frequent in the mefloquine-artesunate group, both in adults and children.

**Conclusion:**

Dihydroartemisinin-piperaquine is as effective as mefloquine-artesunate in treating uncomplicated *P. falciparum* malaria but it is better tolerated and more affordable than mefloquine-artesunate (US$1.0 *versus* US$18.65 on the local market). Therefore, it should be considered as a potential candidate for the first line treatment of *P.falciparum* malaria in Peru.

**Trial Registration:**

ClinicalTrials.gov NCT00373607

## Introduction

In Peru, malaria is one of the most common parasitic diseases and an important public health problem. The highest incidence of *P. falciparum* malaria occurs in the Peruvian Amazon region where the current first line treatment, mefloquine associated with artesunate (MAS3), was introduced in 2001 following high resistance to chloroquine (CQ) and sulfadoxine/pyrimethamine (SP) [1]–[2]. Though highly efficacious against multi-drug resistant *P. falciparum*, MAS3 is not well tolerated and its compliance is poor: 14% of treated patients experienced adverse events in a post-marketing surveillance study carried out in Peru [3]. In addition, MAS3 is extremely expensive for the Peruvian Ministry of Health which fully subsidises its costs at 18.65 USD per adult treatment [4].

Dihydroartemisinin-piperaquine (DHA-PPQ) is a new artemisinin-containing fixed-combination treatment (ACT) that has proved to be well tolerated and highly efficacious against uncomplicated *P. falciparum* malaria in Southeast Asia [5]–[11] and in Africa [12]. Piperaquine, an orally active bisquinoline, is structurally related to CQ. Piperaquine and aminoquinolines such as CQ may have similar targets, possibly the inhibition of the heme-digestion pathway in the parasite food vacuole [13]. Some resistance did develop in China when piperaquine was widely used as monotherapy against CQ-resistant *P. falciparum*. However, its combination with dihydroartemisinin has shown to be active against multi-drug resistant *P. falciparum* [8]. DHA-PPQ is not only much cheaper than MAS3 (1.00 USD *versus* 18.65 USD per adult treatment) [4] but it is also co-formulated, a major advantage favouring higher patient's compliance. DHA-PPQ has been registered in China, Cambodia and recently in Vietnam, where it represents a substantial percentage of the national consumption of anti-malarial treatments [14].

We have carried out the first clinical trial in Latin America evaluating the safety, tolerability and efficacy of DHA-PPQ *vs* the current MAS3 regimen in Peruvian patients with uncomplicated *P. falciparum* malaria and results are reported below.

## Materials and Methods

The protocol for this trial and supporting CONSORT checklist are available as supporting information; see Checklist S1 and Protocol S1.

### Study design, sites and patient treatments

A randomised, open-label clinical trial was carried out between July 2003 and July 2005 in 9 health posts (HP) and 4 commune health centres (CHC) in the rural communities located south of Iquitos, the largest city in the Peruvian Amazon region (population approximately 350,000 inhabitants) [15]. All health facilities draw their patients from the rural areas surrounding the town. Malaria transmission in the area is low with a peak between November and June; the main vector is *Anopheles darlingi* [16]. *Plasmodium falciparum* represents 20%–30% of all malaria infections, the rest being due to *P. vivax*. All age groups are at risk, with the majority of infections progressing towards symptomatic disease, though severe malaria cases are rare [17].

Patients aged 5–60 years old attending the health facilities and with suspected clinical malaria had a blood slide done (thick blood film). If positive for *P. falciparum* they were referred to the San Juan Health Centre where another blood slide was collected. They were enrolled in the study if they met, besides being in the required age range, the following inclusion criteria: fever (axillary temperature≥37.5°C) or history of fever in the preceding 24 h and mono-infection with *P. falciparum* with asexual parasite density between 1,000 and 200,000/µl. Patients were excluded if they had at least one of the following exclusion criteria: severe malaria, pregnancy or lactation, any other concomitant illness or underlying disease, contra-indication to the trial drugs, history of treatment with mefloquine in the last 60 days or CQ, primaquine or quinine within the 14 days before the current episode.

Patients meeting the inclusion/exclusion criteria were enrolled in the study only after written informed consent was obtained from them or their carer. The study protocol was approved by the Ethical Review Committee of the Universidad Peruana Cayetano Heredia and that of the Institute of Tropical Medicine, Antwerp, Belgium. The research was performed in accordance with the ethical standards of the Peruvian Ministry of Health. The trial has been registered as an International Standard Randomised Controlled Trial, number NCT00373607 at http://www.clinicaltrials.gov.

Patients were randomised in blocks of ten to receive either MAS3 or DHA-PPQ. Sealed opaque envelops containing the treatment allocations were opened only after the final decision to recruit the patient had been made. Drugs were administered once daily for 3 days according to the patient's body weight: MQ = 8 mg/kg/day (Lariam^TM^, Hoffman-La Roche Co., Basel, Switzerland) and AS = 4 mg/kg/day (Artesunate^TM^, Guilin Pharmaceutical Factory, Guilin, China), DHA-PPQ = 2.1 mg/kg/day DHA and 16.8 mg/kg/day PPQ (Artekin^TM^; Holleykin pharmaceuticals, Guangzhou, China). The number of pills was rounded off to the nearest quarter tablet. Patients were observed for 1 hour: if vomiting occurred within 30 min of dosing, a full dose was re-administered; if vomiting occurred between 30 min and 1 hour only half dose was re-administered. For young children, drugs were crushed, dissolved in water and squirted into their mouths.

### Treatment follow-up

All enrolled patients were followed up daily until day 3 (24–48–72 h) and were asked to return on days 7, 14, 21, 28, 35, 42, 49, 56 and 63 or outside scheduled visits if any symptom occurred. If the patient did not report for scheduled visits, every effort was made to locate him/her at the home address. During each visit, a clinical assessment was done and a blood sample for parasitaemia taken by finger prick. At Day 0 and at any visit from Day 7 onwards, a blood spot on filter paper was also taken for later PCR analysis on recurrent parasitemia cases. PCV was measured at Day 0, 7 and 14; for those still in the follow up, a last measurement was done at day 63; anaemia was defined as a PCV≤33%. A venous blood sample was taken at D0 and D7 for a complete blood count, liver and renal function tests. Rescue treatment was provided to patients with recurrent parasitaemia or with serious adverse events requiring treatment withdrawal. Patients treated with DHA-PPQ were given MAS3 and those on MAS3 received DHA-PPQ. Severe malaria cases were treated with parenteral quinine. *P.vivax* infections were treated with chloroquine (25 mg/kg over 3 days).

### Clinical and laboratory procedures

Clinical examination systematically assessed body temperature (axillary temperature), heart and respiratory rate, blood pressure, liver and spleen size. All signs and symptoms were systematically recorded on a pre-coded standardized form.

The thick and thin blood films were stained with Giemsa 10% for 10 minutes and reading for species determination and parasite density was blinded to treatment. Parasite density was calculated by counting the number of asexual forms for 200 white blood cells (WBC) and assuming 8,000 WBC/µl. A blood smear was declared negative if no asexual form was detected after reading 100 microscopic fields. If sexual forms were detected, a gametocyte count was performed against 1,000 WBC. Quality control was performed on 10% of all slides (random sample) at the National Hospital of the Cayetano Heredia University in Lima. The agreement between the first and second reading was 98%.

PCV was measured using micro capillary tubes which were centrifuged and PCV determined following the Hawksley micro-haematocrit reading method [18].

A blood spot (2 cm^2^) was taken on filter paper (Whatman n°3) for later PCR analysis. Each PCR filter paper was dried and stored individually in a plastic bag containing silica gel. Parasite genotyping was done by a nested PCR for variable blocks within the merozoite surface protein 1 (MSP1), MSP2 and glutamate-rich protein (GLURP), as described previously [19]–[21]. A recrudescent infection was defined as one that showed match in size of at least one allele for all 3 genes (MSP1, MSP2 and GLURP) between Day 0 and the day of recurrent parasitemia. A new infection was defined when a different genotyping pattern was observed in at least one of the 3 genes, between Day 0 and the day of recurrent parasitemia. When it was not possible to distinguish recrudescence from new infection, the sample was classified as indeterminate.

### Sample size calculation

Assuming a risk of recrudescence adjusted by genotyping of 6% for MAS3 and 1% for DHA-PPQ group, we calculated that a minimum of 250 patients would be needed in each treatment arm to detect a significant difference at 5% significance level and 80% power.

### Study end-points and case definitions

The primary outcome was the PCR-corrected cure rate at day 63. Secondary outcomes were parasite clearance and fever clearance, gametocyte carriage and incidence of adverse events (AE). Treatment outcome was established according to the World Health Organization and Pan American Health Organization guidelines for *in vivo* drug testing [22]. Early treatment failure (ETF) was defined as one of the following: (i) danger signs or severe malaria at Days 1, 2 or 3 with parasitaemia; (ii) parasite density at Day 2>Day 0; (iii) parasitaemia at Day 3 with fever (axillary temperature≥37.5°C); (iv) parasitemia at Day 3≥25% than Day 0. Late clinical failure (LCF) was defined as danger signs, severe malaria or parasitaemia and fever between Days 4 and 63 without having been previously classified as ETF. Late parasitological failure (LPF) was defined as recurrent parasitaemia between Day 4 and 63 without fever and without previously meeting any of the criteria for ETF or LCF. Adequate Clinical and Parasitological Response (ACPR) was defined as no parasitaemia until Day 63 without previously meeting any of the criteria for ETF, LCF, and LPF.

An adverse event (AE) was defined as “any unfavourable and unintended sign, symptom, or disease temporally associated with the use of the drug administered”.

### Statistical analysis

Data were double-entered and cleaned in ACCESS, Microsoft office 2003, and analysed in STATA version 8.0 (Stata Corporation, College Station, Texas, US). Descriptive statistics were used to summarise baseline clinical and biological values of all randomized patients. All PCR indeterminate results were considered as treatment failures, adopting a worst case scenario.

Data were analysed by Intention-to-treat (*ITT*), Per-protocol (*PP*) and Kaplan Meier survival analysis to determine the cure rate (PCR-adjusted) at day 63 in each treatment group. In the ITT analysis, patients lost to follow up were considered as treatment failures (worst case scenario) and the denominators constituted by the patients initially randomized to each group. In the *PP* analysis, patients lost to follow-up and those excluded after enrolment were excluded from the analysis, and only patients with complete follow-up were included in the denominator. In the *ITT* and *PP* analysis, cure rates were compared using chi-square (χ^2^) test with Yates' correction or two-tailed Fisher's exact test as appropriate. In the Kaplan Meier survival analysis, each patient contributed to the analysis for the time s/he was followed up. Patients lost to follow up or with a new Plasmodium infection (*P.falciparum, P.vivax*) were censored at the time they were last seen for the primary outcome. In the survival analysis, cure rates were described by Kaplan Meier estimates and compared between groups with a log-rank test. Gametocyte carriage rates were estimated as proportion of patients with gametocytemia at day 0, 7, 14, etc, until total clearance in both groups, and person-gametocyte-weeks were calculated to measure gametocyte carriage and transmission potential. The person-gametocyte-weeks rates were calculated as the number of weeks in which blood slides were positive for gametocytes during the 2 first weeks of follow-up after treatment divided by the number of all follow-up weeks and expressed per 1,000 person-weeks. The risk of adverse events during the first week after the start of the treatment was calculated for each group separately in children and adults. Relative risks and p-values were computed using chi-square (χ^2^) test with Yates' correction or two-tailed Fisher's exact test as appropriate.

## Results

### Patient's characteristics

**Figure 1:**
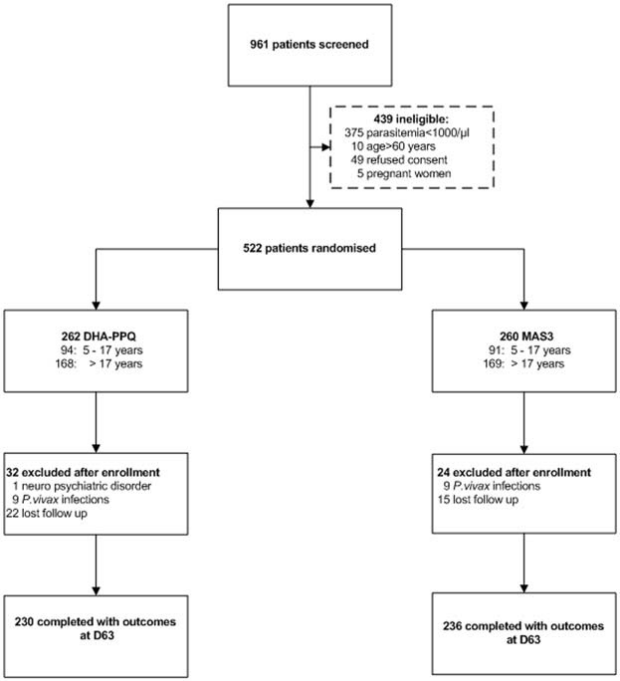
Trial profile.

Out of 961 patients screened at the San Juan Health Centre where all potential study subjects were referred from the surrounding health facilities, 522 were enrolled in the study, 262 randomized to DHA-PPQ and 260 to MAS3 (Figure 1). The main reasons for ineligibility were low parasitemia (85%) and unwillingness to participate because not available for the whole follow-up period (11%). Two third of the patients (65%) were adults (18–60 years old) and the rest children 5–17 year old. Twenty two (8%) patients treated with DHA-PPQ and 15 (6%) with MAS3 were lost to follow up, the main reason being having left the study area because of work (forest workers, etc). These patients were included in the survival analysis as event-free for the time they participated, while in the *ITT* analysis they were considered as failures, and in the *PP* they were excluded from the analysis. Baseline characteristics were comparable in the 2 groups, except for gametocytaemia on admission which was slightly higher in MAS3 (17%) than in the DHA-PPQ (13%) group (p = 0.31). The median PCV at day 0 was comparable in both treatment groups (Table 1). No difference was detected at day 7 (36% for DHA-PPQ and 35% for MAS3) and day 14 (35% in both groups). At day 63, median PCV was 38% in both groups, a similar value than that at day 0. The prevalence of anaemia was 24% for the DHA-PPQ and 20% for the MAS3 group at day 0, 30% in both groups at day 14 and 15% for DHA-PPQ and 10%, for MAS3 at day 63.

**Table 1:** Baseline characteristics of all randomized patients by treatment group.

CHARACTERISTICS	DHA-PPQ	MAS3
**Number of patients**	262	260
**Sex ratio (Male/Female)**	1.4 (152/110)	1.6 (161/99)
**Age groups (years)**		
**5–17**	94 (36%)	91 (35%)
**>17**	168 (64%)	169 (65%)
**Body weight (Kg), median [Q1–Q3]**	52.5 [38.8–60.2]	51.9 [39.8–59.4]
**Geometric mean parasite count/µL [95%CI]**	5,866 [5,214; 6,600]	5,304 [4,731; 5,946]
**Gametocytaemia on admission**	35 (13%)	43 (17%)
**Axillary temperature≥37.5°C**	137 (52%)	126 (48%)
**Haemoglobin (g/dl), mean±SD**	12.2±1.7	12.2±1.6
**PCV (%), median [Q1–Q3]** °	38 [34–41]	37 [34–40]

° [Q1–Q3] = inter-quartile range = [Lower–Upper Quartile].

No patient had abnormal liver and renal function test results of clinical significance, both at entry and at day 7 (data not shown).

### Clinical and parasitological outcomes

Twenty-four hours after the first dose, a significantly higher proportion of patients in the DHA-PPQ group had cleared parasitaemia [67.7% *versus* 58.5%, p = 0.03], though this difference tended to disappear at day 2 so that by day 3 all patients had cleared parasitaemia (Table 2). Fever clearance was equally high, at day 2 almost all patients were afebrile, with no difference between the 2 groups (Table 2).

**Table 2:** Clinical and parasitological outcomes by treatment group.

RESULTS	DHA-PPQ	MAS3	p-value
Parasite clearance at 24 hours	67.7% (176/260)	58.5% (152/260)	0.03
“ “ at 48 hours	98.9% (257/260)	96.2% (250/260)	0.05
Fever clearance at 24 hours	88.9% (232/261)	88.1% (229/260)	0.77
“ “ at 48 hours	98.5% (255/259)	99.6% (259/260)	0.18
**ETF**	**0**	**0**	
Total recurrent parasitemia day 4–63	10% (23/230)	5% (12/236)	0.07
- *P.vivax infections*	11	10	0.07
- New *P.falciparum* infections	8	1	
- Late Parasitological Failure-PCR corrected	4	1*	
**63-day cure rate (ACPR-PCR adjusted)**			
*- Intention-to-treat*	90.1% (236/262)	93.8% (244/260)	0.15
*- Per protocol*	98.3% (226/230)	99.6% (235/236)	0.21
*- Kaplan-Meier survival analysis*	98.4%	99.6%	0.18°

**ETF** = Early Treatment Failure; **ACPR** = Adequate Clinical and Parasitological Failure; * PCR indeterminate classified recrudescence;° Logrank test.

No ETF was observed. Between Day 4 and 63, 23 patients (10%) in the DHA-PPQ and 12 (5%) in MAS3 group had recurrent parasitemia, among which *P. vivax* represented 11 and 10 infections, respectively (Table 2). In the DHA-PPQ group and after PCR genotyping, 8 of the 12 recurrent falciparum infections were classified as new infection and 4 as recrudescence (at day 13, 25, 42, and 63). In the MAS3 group, there was one new infection and 1 with indeterminate PCR result that was classified as recrudescence (at day 37). All recrudescences were classified as LPF.

The PCR-corrected ACPR obtained by survival analysis was 98.4% [95% CI: 96.7–99.4] in the DHA-PPQ group and 99.6% [95% CI: 97.2–99.9] in the MAS3 group (log rank p = 0.18). Results were very similar in the *PP analysis,* 98.3% *vs* 99.6% (RR: 0.99, 95%CI [0.97−1.01], Fisher Exact p = 0.21). In the *ITT analysis,* the ACPR was obviously lower than the other estimates, 90.1% [95%CI: 85.8–93.4] for DHA-PPQ and 93.8% [95%CI: 90.2–96.4] for MAS3, but not significantly different (RR: 0.98, 95%CI [0.86−1.11], χ^2^ p = 0.79].

**Figure 2:**
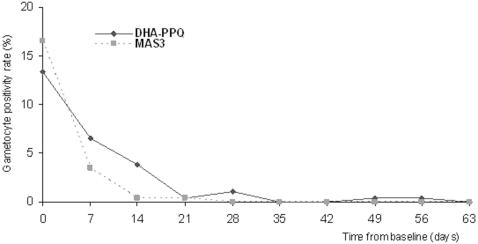
Proportions of patients with gametocytemia during the follow up.

Before treatment, 35 patients (13%) in DHA-PPQ and 43 patients (17%) in the MAS3 group had gametocytaemia. After treatment, the gametocyte carriage rate decreased faster in MAS3 (gametocytes cleared by day 28) than in the DHA-PPQ group (gametocytes cleared by day 35) (Figure 2). Among patients with gametocytes at day 0, gametocyte clearance at day 7 was similar in the 2 treatment groups (73.5%, 25/34 for DHA-PPQ and 84.0%, 36/43 for MAS3; p = 0.27) while it was significantly different at day 14 (73.5%, 25/34 for DHA-PPQ and 98.0%, 41/42 for MAS3; p = 0.002). Among patients without gametocytes at day 0, 8 (3.6%) in the DHA-PPQ group and 2 (0.9%) in the MAS3 group had gametocytes in at least one blood slide between day 0 and 7 (RR: 3.84, 95%CI [0.82–17.87]); no new cases of gametocytaemia were detected between day 7 and day 14. Gametocyte carriage measured as person-gametocyte-weeks was higher but not significantly different in the DHA-PPQ (32.5/1000) than in MAS3 group (24.9/1000) (p = 0.31) (Table 3).

**Table 3:** Gametocyte prevalence and incidence by treatment group.

	DHA-PPQ	MAS3	p-value
**Gametocyte prevalence**			
Day 0	35/262 (13.4%)	43/260 (16.5%)	0.31
Day 7	17/256 (6.6%)	9/256 (3.5%)	0.11
Day 14	10/253 (4.0%)	1/253 (0.4%)	0.006
Day 21	1/247 (0.4%)	1/250 (0.4%)	0.99
Day 28	3/243 (1.2%)	0/249	0.08
Day 35	0/243	0/245	
**Gametocytes incidence**			
Day 7	8/222 (3.6%)	2/213 (0.9%)	0.06
Day 14	0/209	0/208	
**Person-gametocyte-weeks** (/1000 person-weeks)	32.5 (71/2182)	24.9 (55/2207)	0.31

### Adverse events

Overall during the first week of treatment, adverse events were more frequent among patients treated with MAS3 compared to those treated with DHA-PPQ (Table 4), and they were also more severe in the former group (data not shown). In adults as well as in children, dizziness and nausea were the most common reported adverse events. In adults treated with DHA-PPQ, dizziness, nausea and vomiting were significantly less frequent than in those treated with MAS3. Similarly, although insomnia and anxiety were less frequent, they occurred significantly less often in adults treated with DHA-PPQ (p<0.001). Somnolence was rare and not observed in the MAS3 group. Among children, the higher frequency of adverse events in the MAS3 group was significant only for insomnia and anxiety. Vomiting within one hour of treatment intake occurred rarely and with the same frequency in both groups (4%: 10 patients in the DHA-PPQ and 11 in the MAS3 group).

**Table 4:** Adverse events by age and treatment group within the first week of follow up.

	ADULTS				CHILDREN			
	DHA-PPQ	MAS3	Relative Risk	p	DHA-PPQ	MAS3	Relative Risk	p-value
**Nausea**	77/161 (48%)	100/164 (61%)	0.78	0.02	49/101 (49%)	56/96 (58%)	0.83	0.17
**Vomiting**	29/161 (18%)	48/164 (29%)	0.62	0.02	24/101 (24%)	25/96 (26%)	0.91	0.71
**Dizziness**	122/161 (76%)	152/164 (93%)	0.82	<0.001	63/101 (62%)	66/96 (69%)	0.91	0.35
**Anorexia**	69/161 (43%)	77/164 (47%)	0.91	0.46	42/101 (42%)	35/96 (36%)	1.14	0.46
**Abdominal pain**	42/161(26%)	45/164(27%)	0.95	0.78	31/101 (31%)	34/96 (35%)	0.87	0.48
**Insomnia**	22/161 (14%)	71/164 (43%)	0.32	<0.001	5/101 (0.5%)	27/96 (28%)	0.18	<0.001
**Somnolence**	5/161 (3%)	0/164	.	0.02	3/101 (2.3%)	0/96	.	0.09
**Anxiety**	3/161 (1.2%)	24 (15%)	0.13	<0.001	0/101	5/96 (5%)	0	0.02

The majority of adverse events were transient and resolved spontaneously with the only exception of a woman who complained of anxiety and insomnia during the whole follow-up period and needed the administration of sedatives and anxiolytic drugs. Serious drug-related adverse events requiring early treatment termination occurred in 3 patients treated with MAS3. A 30 years old man suddenly presented at day 4 with severe dizziness, loss of balance, unsteadiness, amnesia and severe anxiety. Immediately after the end of the treatment, he reported severe headache and insomnia. Clinical and biological examinations diagnosed a toxic metabolic encephalopathy, possibly due to MAS3. A 41 years old man had severe weakness, anxiety, and transient arrhythmia 30 minutes after the second dose of MAS3. The third patient, a 6 years old boy, presented with palpitations and chest pain, 12 hours after the second dose of MAS3.

## Discussion

This first clinical trial carried out in South America on DHA-PPQ showed that this new fixed-dose ACT is as efficacious as MAS3 but better tolerated and with significantly less adverse events. These excellent results strongly support DHA-PPQ as a good alternative to MAS3, the current first line treatment in the Peruvian Amazon region. Moreover, compared to MAS3, DHA-PPQ is less expensive (1.00 USD *versus* 18.65 USD per adult treatment) [4], a major advantage for the Peruvian health authorities that fully subsidise antimalarial treatment. Cure rates at the end of the follow up were extremely high for both treatment groups, regardless of the analysis method, though MAS3 tended to have higher cure rates. Our results are conservative, since we opted for the worst case scenario and considered indeterminate PCR results as recrudescence. In the best case scenario, the 63 day cure rate would have been 100% in the MAS3 group. Our results are similar to those reported for DHA-PPQ from Southeast Asia: Thailand (63-day cure rate 96.1% [7]), Burma (42-day cure rate 99.4% [10]), Cambodia (28-day cure rate 96.9% [6], 63-day cure rate 97.5% [11]), Vietnam (56-day cure rate 98.7% [8]); and from a recent study in Rwanda (28-day cure rate 95.2% [12]).

Most patients lost to follow up were forest workers who had to go and work outside the study area. It is impossible to know whether they experienced a recurrent infection within the 63 days after treatment but it is unlikely that all of them had a recrudescence. However, all of them were considered as failures in that ITT analysis, usually recommended in superiority trials to avoid potential bias introduced by patients lost to follow-up [23]. This is why the ACPR computed by *ITT* analysis is likely to be an underestimation of the true cure rate.

Though patients in the MAS3 group had a slightly higher prevalence of gametocytemia before treatment, they cleared it more rapidly than patients in the DHA-PPQ group. The latter had a higher person-gametocyte-weeks rate but the difference was not significant compared to MAS3. New gametocytes appeared more frequently during the first week in patients treated with DHA-PPQ than those treated with MAS3, but these small differences were not found significant with the power of our study. As mentioned previously, the lower dose of artemisinin contained in DHA-PPQ as compared to MAS3 may be responsible of these differences [7], [10].

The incidence of new infections was higher in the DHA-PPQ than in the MAS3 group, possibly reflecting the shorter post-treatment prophylactic effect of piperaquine as compared with mefloquine. It should be noted that all new infections in the DHA-PPQ group occurred at least one month after treatment, from day 35 onwards (most of them, six out of 8, by day 49 or later), while in the MAS3 group the only new infection detected occurred at day 21. However, a similar study (63-day follow up) carried out in Cambodia in a low transmission area and comparing DHA-PPQ with MAS3 did not report any difference in the incidence of new infections [11].

The occurrence of adverse events was generally lower and less severe in patients treated with DHA-PPQ than in those treated with MAS3, both in adults and children. Their high occurrence in the MAS3 group differ considerably from that observed in another trial carried out in Peru in 2000 [24], in which very few cases of vomiting and insomnia were reported, possibly because of the lower dose of MQ (15 mg/kg) administered. Since our study was not blinded, responder bias cannot be ruled out: patients in the MAS3 group, especially adults, were more likely to report adverse events (especially gastro-intestinal) since some of them may have had previous negative experiences with this antimalarial treatment. Nevertheless, most adverse events observed in our patients are known to occur with MAS3 and their frequency was similar to that reported in previous trials [8]–[11]. Neuro-psychiatric events, such as anxiety and insomnia, were much less frequent in the DHA-PPQ group, and the difference was significant both in adults and children. It is difficult to say whether this is a true difference or due to a responder bias, both for adults and for the children's parents/caretakers. Nevertheless, the type and frequency of adverse events reported is consistent in both age groups and indicate a lower tolerability of MAS3 as compared to DHA-PPQ.

In conclusion, our study confirmed that DHA-PPQ is a highly effective antimalarial treatment for *P. falciparum* malaria in the Amazon region of Peru. This co-formulation is safe and better tolerated than MAS3 and thus may have a better compliance. All these advantages combined to its low cost makes DHA-PPQ a suitable and even advisable first line treatment for *P. falciparum* malaria in Peru. Our encouraging results should prompt neighbouring endemic countries to conduct similar efficacy trials, and possibly change their anti-malaria treatment policy to this highly efficacious, safe, well tolerated and affordable ACT.

## Supporting Information

Protocol S1 Trial Protocol
(0.13 MB DOC)

Checklist S1 CONSORT Checklist
(0.05 MB DOC)
