# Supplementary material for: A Randomised Controlled Trial to Assess the Efficacy of Dihydroartemisinin-Piperaquine for the Treatment of Uncomplicated Falciparum Malaria in Peru
Source: PLoS One. 2007 Oct 31;2(10):e1101. doi: 10.1371/journal.pone.0001101 (PMC2040506; doi:10.1371/journal.pone.0001101)
Supplement: Protocol S1 — Trial Protocol (0.13 MB DOC) [file pone.0001101.s001.doc]

A RANDOMISED OPEN HOSPITAL-BASED STUDY TO ASSESS THE SAFETY AND EFFICACY OF DIHYDROARTEMISININ-PIPERAQUINE (ARTEKIN) FOR THE TREATMENT OF UNCOMPLICATED FALCIPARUM MALARIA IN IQUITOS, PERU

***Study drugs***: Artekin (dihydroartemisinin+piperaquine) vs Mefloquine+artesunate

***Design***: Randomised, open trial with 2 arms

***Study site***: Regional Hospital, Iquitos, Peru

***Authors*** U. D’Alessandro

1. Llanos

JC. Dujardin

C. Miranda

D. Gamboa

J. Arevalo

***External Advisory Committee***: F. Nosten, Liz Ashley, Prof NJ White

1. RATIONALE AND BACKGROUND

Artekin is a new antimalarial drug combination containing piperaquine and

dihydroartemisinin (DHA). Piperaquine is a bisquinoline compound developed by the

Chinese with good activity against multidrug resistant *Plasmodium falciparum* malaria.

The first human studies were carried out in the 1970s and involved its prophylactic use

in several thousand adults and children. In China piperaquine has been in use as

monotherapy for over twenty years. It has proved generally very well tolerated and

effective. More recently, piperaquine has been used as a part of short-course

artemisinin-based combination oral therapies designed to have a high cure rate and few

side effects. The first of such combinations was China-Vietnam 8 (CV8) and included

DHA, piperaquine, primaquine and trimethoprim. Although CV8 was effective, the

role of trimethoprim as an antimalarial drug was questionable and the risk of

primaquine-induced hemolysis in individuals with glucose-6-phosphate dehydrogenase

deficiency was cause of concern. Moreover, the total DHA dose in a course of CV8

given under the manufacturer’s recommendations was lower than that given in the form

of DHA monotherapy. Therefore, trimethoprim and primaquine have been eliminated

from the combination Artekin, whose DHA content is higher than that of CV8. DHA

has already been extensively evaluated and shown to be safe – with no identifiable

adverse effects.

Several factors make this combination a very exciting prospect for the future. This is a

fixed dose co-formulation that will surely improve the patient’s compliance. It is

registered in Cambodia, China and Vietnam that has decided that Artekin should

represent one half of all antimalarial treatments used in the country. Therefore, Artekin

may become the most widely used combination in South East Asia. Moreover, Artekin

is inexpensive in comparison with other combinations such as mefloquine-artesunate,

the first line drug in Iquitos, and it costs approximately 1US$ per adult treatment.

Finally, preliminary clinical trials in small groups of patients have shown that Artekin is

well tolerated and highly effective. A recently published non-randomised trial carried

out in Cambodia on 106 malaria patients (76 children and 30 adults) reports a cure rate

of 96.6% (98.6% in children and 92.3% in adults) at day 28 with 21% of the patients

reporting minor side effects (Denis et al 2002).

This study will be linked to similar studies in Thailand, Cambodia and

Vietnam with the aim of accumulating data on safety, efficacy and toxicity of

Artekin in adults and children. If Artekin confirms its safety, tolerability and efficacy, it

could replace the current first line treatment in Iquitos. It will decrease the treatment

cost and improve patient’s compliance.

2. OBJECTIVES:

The general objective of this study is to evaluate the new combination Artekin for the treatment of patients with uncomplicated malaria.

Specific objectives are the following:

1. To compare the safety, tolerability and efficacy of dihydroartemisinin-piperaquine to that of the mefloquine-artesunate 3-day regimen (MAS3) in patients with uncomplicated *falciparum* malaria;
2. To study the pharmacokinetic of piperaquine and mefloquine by means of a population-based study;

3. STUDY DESIGN:

This is a randomised open study comparing Artekin and MAS3 for the treatment of symptomatic uncomplicated *falciparum* malaria. The differences in drug formulations mean that blinding when treatment is administered is not possible. Nevertheless, the laboratory technicians reading all malaria smears will have no knowledge of the treatment received by individual patients.

*3.1 Recruitment*

Patients attending the Iquitos regional hospital and suspected of having a clinical attack of malaria will be included in the study if they fulfil the following criteria:

### Inclusion criteria:

- Age: 5-60 years old;
- Fever (body temperature≥37.5°C) or history of fever in the previous 24 hours;
- Monoinfection with *P.falciparum* with parasite density between 1,000-200,000/µl
- Written informed consent to participate to the trial. For patients aged less than 18 years, an informed consent will be obtained from a parent or a guardian.

Exclusion criteria:

- Mixed malaria infection
- Pregnancy or lactation (urine test for β HCG to be performed on any woman of child bearing age unless menstruating).
- Concomitant illness
- Danger signs
- not able to drink
- incontrollable vomiting
- recent history of convulsions (>1 in 24 hours)
- unconscious state; neurological impairment
- unable to sit or stand
- Signs of severe malaria

1. Cerebral malaria (unrousable coma)
2. Severe anaemia (Htc< 15%)
3. Renal failure (serum creatinine > 3 mg/dL)
4. Pulmonary oedema
5. Hypoglycemia (<40mg/dL)
6. Shock (systolic BP < 70 mmHg in adults, 50 in children)
7. Spontaneous bleeding/DIC
8. Repeat generalized convulsions
9. Macroscopic haemoglobinuria
10. Severe jaundice

- History of mefloquine treatment within 60 days before the current episode
- History of convulsions or neuropsychiatric disorder.
- Known hypersensitivity to artemisinins or mefloquine- defined as history of erythroderma/other severe skin reaction, angioedema or anaphylaxis.
- History of splenectomy

*3.2 Treatment allocation*

Patients will be randomly allocated to receive one of 2 treatments, i.e. Artekin or MAS3. There will be 2 lists, one for patients aged <18 years and the other for the adults. In each list the randomisation will be in blocks of 10 numbers, each of them corresponding to a treatment. Patients will be assigned a number as they are recruited and will receive the corresponding treatment according to the randomisation list.

*3.3 Clinical procedures at day 0*

All subjects enrolled in the study will be given a unique code corresponding to the randomisation list. A case-record form will be completed for each patient documenting symptoms prior to clinic attendance, concomitant illness, drug history.

A physical examination will be recorded. A venous blood sample will be taken on admission for complete blood count, creatinine and liver function tests. These investigations will be repeated on day 7. A blood spot for parasite genotyping will be collected on filter paper to be stored in individual sealed bags with desiccant.

*3.4 Drugs and dosage*

Treatment will be administered for 3 days under the direct supervision of health workers under supervision of a licensed physician. The correct dose will be determined according to the weight dosing chart (Table 1-2). For young children, study drugs (either whole or as fractions) will be crushed, dissolved in water and squirted into their mouths using a syringe. After drug administration, patients will be observed for one hour in the clinic. A dose will be repeated in full if vomiting occurs within 30 minutes of administration and halved if vomiting is between 30 minutes and 1 hour post dosing. This event will be documented in the case record form (CRF).

i) Mefloquine + Artesunate (MAS3)

The MAS3 regimen is artesunate 4 mg/kg/day once daily for 3 days plus mefloquine 24 mg/kg given as a three day regimen of 8mg/kg/day (Table 1).

ii)Dihydroartemisin-piperaquine (*Artekin*, Hualijian Pharmaceutical Co. Ltd., Guangzhou, China)

Each tablet contains 40mg of dihydroartemisinin and 320mg piperaquine (Table 2).

Table 1. Daily dose of mefloquine (MQ) (250mg tablets) and artesunate (AS) (50mg tablets)

| **Weight (kg)** | **MQ tablets** | **AS tablets** | **MQ Dose mg/kg/day** | **AS Dose**  **mg/kg/day** |
| --- | --- | --- | --- | --- |
| **15-17** | 1/2 | 1+1/4 | 7.4-8.3 | 3.7-4.2 |
| **18-25** | 3/4 | 1+1/2 | 7.5-10.4 | 3.0-4.1 |
| **26-34** | 1 | 2 | 7.4-9.6 | 2.94-3.8 |
| **35-43** | 1+ 1/4 | 3 | 7.3-8.9 | 3.5-4.3 |
| **44-50** | 1+ 1/2 | 4 | 7.5-8.5 | 4.0-4.5 |
| **51-58** | 1+3/4 | 4+1/4 | 7.5- 8.6 | 3.7-4.2 |
| **59-66** | 2 | 4+3/4 | 7.6-8.5 | 3.6-4.0 |
| **67-72** | 2+1/4 | 5+1/2 | 7.8-8.4 | 3.8-4.1 |
| **73-83** | 2+1/2 | 6 | 7.5-8.6 | 3.6-4.1 |
| **84-90** | 2 + 3/4 | 7 | 7.6-8.2 | 3.9-4.2 |
| **>90** | 3 | 7+1/2 | 8.3- | 4.1 |

Table2. Daily dose of Artekin (40mg DHA and 320mg Piperaquine )

| **Weight (kg)** | **Daily dose**  **Artekin** | **DHA**  **Dose range**  **mg/kg/day** | **Piperaquine**  **Dose range**  **mg/kg/day** |
| --- | --- | --- | --- |
| 13-20 | 1 | 1.7-2.5 | 13.3-20.0 |
| 21-30 | 1+1/2 | 2.0-2.9 | 12.0-22.9 |
| 31-40 | 2 | 2.0-2.58 | 16.0-20.1 |
| 41-50 | 2+1/2 | 2.0-2.4 | 16.0-19.5 |
| 51-60 | 3 | 2.0-2.4 | 16.0-18.8 |
| 61-70 | 3+1/2 | 2.0-2.3 | 16.0-18.3 |
| 71-84 | 4 | 1.9-2.3 | 15.2-18.0 |
| 85-100 | 5 | 2.0-2.35 | 16.0-18.8 |

*3.5 Follow-up*

Patients or their parent/guardian will be asked to return to the hospital 24 and 48 hours later for drug administration and for scheduled tests at 72 hours, days 7, 14, 21, 28, 35, 42, 49, 56 and 63. If the patient does not report for scheduled visits every effort will be taken by the nurses to locate him/her at his/her home address. Patients will be encouraged to attend the hospital outside a scheduled visit whenever they feel sick. At each visit, the history, the clinical signs and symptoms, the body temperature and a blood sample for parasitaemia will be collected. A blood spot on filter paper for the molecular analysis (MSP1 and MSP2 for the identification of new infections) will also be collected at each visit from day 14 onwards. Hematocrit will be measured at day 0 and 14 while creatinine and liver function tests at day 0 and Day 7 (Table 3).

**Table 3. Follow-up**

| **Day** | **0** | **1** | **2** | **3** | **4** | **5** | **6** | **7** |  | **14** | **21-63** |
| --- | --- | --- | --- | --- | --- | --- | --- | --- | --- | --- | --- |
| Treatment | X | X | X |  |  |  |  |  |  |  |  |
| History | X |  |  |  |  |  |  | X |  | X | X |
| Examination (clinical) | X | X | X | X |  |  |  | X |  | X | X |
| Temperature | X | X | X | X |  |  |  | X |  | X | X |
| Blood film | X |  | X | X |  |  |  | X |  | X | X |
| Filter paper PCR | X |  |  |  |  |  |  |  |  | X | X |
| Filter paper PK* | X | X | X | X |  |  |  | X |  | X | X |
| Haematology | X |  |  |  |  |  |  |  |  | X |  |
| Adverse drug reactions |  | X | X | X |  |  |  | X |  | X | X |
| Biochemistry | X |  |  |  |  |  |  | X |  |  |  |

* until day 28 included

Blood samples

*Haematology*: PCV (Ht), WBC total and differential counts

*Blood films*: Duplicate thick and thin blood smears will be taken at enrolment (Day 0) and every day until negative. Subsequently, a blood slide will be done on any other visit (scheduled and non-scheduled). After fixing the thin film in methanol, both smears will be Giemsa-stained, pH 7.2 and the thick film examined under oil-immersion for malaria parasites. *P. falciparum* parasites will be counted against 200 white blood cells (WBC). Assuming the median total WBC count of 8,000/µl for the study population, each parasite count will be multiplied by forty (x40) to quantify the parasite density per μl of blood.

Parasite density per µl = number of parasites x WBC count (8000) *

number WBC counted (200)

A smear will be declared negative after examination of 100 microscopic fields. If >500 parasites have been counted without having reached 200 WBC, the count is stopped after completing the reading of the last field and the parasitaemia is calculated according to formula above. If *P.falciparum* gametocytes are seen, a gametocyte count is performed against 1000 WBC.

*Genotyping*: A filter paper blood specimen will be collected at day 0 before treatment, on day 14 and any day thereafter up to day 56. The filter papers will be stored at room temperature under dry conditions until the conclusion of the study, when they will be sent to a suitable laboratory for PCR. Molecular analysis will be done only on filter paper samples from patients who are microscopically positive.

*3.6 Pharmacokinetic studies*

The primary objective of the population kinetic study will be to collect data points from large numbers of patients that will allow us to define the disposition of the drugs when administered in combination and the disease effect. A blood sample for the pharmacokinetic of piperaquine and mefloquine will be collected at randomly allocated times between day 3 and day 28 from the first 200 patients. Blood samples for piperaquine will be stored at –80°C. Blood samples for MQ will be collected in capillary tubes and stored at –20°C. The randomisation schedule for the time of collection will be specified later.

*3.7 Efficacy definitions: Classification of therapeutic response*

*Early treatment failure (ETF)*

- Patient develops danger signs (see above in the exclusion criteria) or severe malaria on days 1, 2 or 3 with parasitaemia.
- Axillary temperature >=37.5^o^C on day 2 and parasite density greater than that at day 0.
- Axillary temperature >=37.5^o^C on day 3 with parasitaemia.
- Parasite density at day 3 equal or greater than 25% of the parasite density at day 0.

*Late treatment failure (LTF)*

This includes late clinical failure (LCF) and late parasitological failure (LPF)

LCF is defined as follows:

- Patient develops danger signs (see above in the exclusion criteria) or severe malaria with parasitaemia between day 4 and day 63.
- Axillary temperature >=37.5^o^C and parasitaemia between day 4 and 63 without having been previously classified as ETF.

LPF is defined as follows:

- Parasitaemia by day 63 and an axillary temperature <37.5°C without previously meeting any of the criteria for ETF or LCF.

*Adequate clinical and parasitological response (ACPR):*

- Absence of parasitaemia by day 63 without previously meeting any of the criteria for ETF or LTF.

Only the a recurring parasitaemia that is confirmed by PCR (and the indeterminate) to be recrudescence will be counted as treatment failure.

The incidence of adverse experiences will be tabulated.

*3.8 Indications for early termination and rescue treatment*

***Reasons for treatment discontinuation***

If at any time the progress of the patient is unsatisfactory he/she or the child’s parent/guardians will be instructed to report immediately to the hospital. Objective criteria for discontinuation from study medication and institution of rescue treatment are:

- Parasitaemia increased > 25% from baseline and no clinical improvement at 48h.
- Any sign of severe malaria, as per WHO definition, or a requirement for parenteral treatment.
- Any severe adverse event requiring treatment withdrawal in the treating physician’s opinion or at the patient’s or parent/guardian’s request
- Patient’s or parent/guardian’s decision

***Rescue treatment***

Patients previously treated with Artekin will be treated with MAS3 if there is no contraindication and those who received MAS3 will receive Artekin. Severe malaria cases will be treated with parenteral quinine.

***Patient withdrawal***

Once a patient is treated as a treatment failure, he/she will be withdrawn from the study. All protocol investigations up to and including the day of treatment failure will be done. Thereafter, the patient will not have other protocol investigations done.

4. SAMPLE SIZE

Both treatment regimen are likely to be extremely effective and there will be little difference in the efficacy of the treatment between the 2 groups of patients. However, the percentage of adverse events and the tolerability might be significantly different between the 2 groups. Assuming a parasitological failure of 1% for Artekin and of 6% for MAS3, 250 patients per arm would be able to detect a significant difference at the 5% level and with 80% power.

**5. STATISTICAL ANALYSIS:**

The analysis of the cure rates at day 28 and at day 63 will be performed both for the intention-to-treat (ITT) patient population (all randomised patients) and the evaluable patient population. The ITT analysis also includes patients who discontinue before the end of the follow-up. These patients will be counted in the ITT analysis as treatment failures regardless of their reason for discontinuation.

Safety and tolerability measurements will be summarised for the ITT patient population.

The pharmacokinetic data collected will be used to construct a population pharmacokinetic model for the drug as described previously (White et al 1999, Simpson et al 1999).

6. EHICAL CONSIDERATIONS

The study will be conducted according to the Helsinki Declaration on the use of human subjects in research. The treatment MAS3 is the first line standard treatment in the region where the study will be carried out. Artekin is a new combination, but its drug components (DHA and piperaquine), are well known and have been administered to thousands of Asian patients. They are well tolerated and their use has not been associated with major adverse events. An informed written consent will be asked to all patients to be enrolled in the trials before being treated for clinical malaria. Patients developing signs of severe malaria will be offered appropriate treatment. Patients developing anaemia or any other disease during the follow up will be treated accordingly.

This protocol will be submitted to the ethical committee of the Universidad Peruana Cayetano Heredia, Lima, Peru, and to the ethical committee of the Prince Leopold Institute of Tropical Medicine, Antwerp; Belgium.

**References:**

Denis MB, Davis TM, Hewitt S, Incardona S, Nimol K, Handeur T, Poravuth Y, Lim C, Socheat D. Efficacy and safety of dihydroartemisinin-piperaquine(artekin) in Cambodian children and adults with uncomplicated falciparum malaria. *Clin. Infect. Dis.* 2002; **35**:1469-76.

Documentation of Artecom for new drug registration. Tropical Medicine Institute, Guangzhou University of Traditional Chinese Medicine, PR China.

Simpson JA, Aarons L, White NJ. How can we do pharmacokinetic studies in the tropics? Trans.R.Soc.Trop.Med..Hyg. 2001; **95**: 347-51.

Roll back Malaria regional evaluation Cambodia; 24/25 January 2002-02-05.

White NJ. Antimalarial drug resistance and combination chemotherapy. *Phil Trans R Soc Lond B*, 1999; **354**:739-749.

White NJ, van Vugt M, Ezzet F. Clinical pharmacokinetics and pharmacodynamics and pharmacodynamics of artemether-lumefantrine. *Clin. Pharmacokinet*. 1999; **37**: 105-25.
